# Spatiotemporal regulation of hydrogen sulfide signaling in the kidney

**DOI:** 10.1016/j.redox.2021.101961

**Published:** 2021-04-02

**Authors:** Maurits Roorda, Jan Lj Miljkovic, Harry van Goor, Robert H. Henning, Hjalmar R. Bouma

**Affiliations:** a Department of Clinical Pharmacy and Pharmacology, University of Groningen, University Medical Center Groningen, Groningen, the Netherlands; b Department of Medical Oncology, University of Groningen, University Medical Center Groningen, Groningen, the Netherlands; c Mitochondrial Biology Unit, Medical Research Council, University of Cambridge, Cambridge, United Kingdom; d Department of Pathology and Medical Biology, University Medical Center Groningen and University of Groningen, the Netherlands; e Department of Internal Medicine, University of Groningen, University Medical Center Groningen, Groningen, the Netherlands

**Keywords:** Hydrogen sulfide, Gasotransmitter, Kidney, Persulfidation, Ischemia-reperfusion injury, Hypoxia

## Abstract

Hydrogen sulfide (H_2_S) has long been recognized as a putrid, toxic gas. However, as a result of intensive biochemical research in the past two decades, H_2_S is now considered to be the third gasotransmitter alongside nitric oxide (NO) and carbon monoxide (CO) in mammalian systems. H_2_S-producing enzymes are expressed in all organs, playing an important role in their physiology. In the kidney, H_2_S is a critical regulator of vascular and cellular function, although the mechanisms that affect (sub)cellular levels of H_2_S are not precisely understood. H_2_S modulates systemic and renal blood flow, glomerular filtration rate and the renin-angiotensin axis through direct inhibition of nitric oxide synthesis. Further, H_2_S affects cellular function by modulating protein activity via post-translational protein modification: a process termed persulfidation. Persulfidation modulates protein activity, protein localization and protein-protein interactions. Additionally, acute kidney injury (AKI) due to mitochondrial dysfunction, which occurs during hypoxia or ischemia-reperfusion (IR), is attenuated by H_2_S. H_2_S enhances ATP production, prevents damage due to free radicals and regulates endoplasmic reticulum stress during IR. In this review, we discuss current insights in the (sub)cellular regulation of H_2_S anabolism, retention and catabolism, with relevance to spatiotemporal regulation of renal H_2_S levels. Together, H_2_S is a versatile gasotransmitter with pleiotropic effects on renal function and offers protection against AKI. Unraveling the mechanisms that modulate (sub)cellular signaling of H_2_S not only expands fundamental insight in the regulation of functional effects mediated by H_2_S, but can also provide novel therapeutic targets to prevent kidney injury due to hypoxic or ischemic injury.

## 1 Introduction

Representing the simplest sulfur-containing molecule, hydrogen sulfide (H_2_S) - also known as sulfane (according to recent nomenclature) [1] - is a flammable colorless gas that has been mainly recognized as a toxic compound. Toxicity occurs already at low concentrations, upon prolonged exposure to concentrations above 2–5 parts per million or acute exposure to 100 parts per million or higher. Major toxicity of H_2_S occurs through inhibition of mitochondrial cytochrome *c* oxidase, leading to metabolic acidosis associated with cardiovascular and respiratory collapse and sudden loss of consciousness [2]. Endogenous H_2_S synthesis in mammalian cells however, produces much lower, non-toxic concentrations and is recognized to have important physiological functions. H_2_S is enzymatically synthesized by cystathionine-β-synthase (CBS) [3], cystathionine γ-lyase (CSE), 3-mercaptopyruvate sulfurtransferase (3-MST) and indirectly by d-amino acid oxidase (DAO) [[3], [4], [5]]. Following nitric oxide (NO) and carbon monoxide (CO), H_2_S was recently recognized as the third gasotransmitter: signaling molecules that can freely diffuse through membranes to transmit information [[5], [6], [7], [8]]. Given the pleiotropic effect of H_2_S on different critical physiological pathways, spatiotemporal regulation of H_2_S is paramount to allow cellular target specificity [9].

H_2_S plays an important role in renal physiology by modulating renal blood flow, endocrine function and metabolism. First, the concentration-dependent vasoactive properties of H_2_S are of major influence on renal blood flow [10,11]. In addition, H_2_S affects renal endocrine function through regulation of renin and angiotensin II receptor levels, and induction of norepinephrine and aldosterone release, as demonstrated in a murine model of heart failure [12]. Further, H_2_S can attenuate ischemia/reperfusion injury through reduction of oxidative stress, which is illustrated by the observation that mice lacking either CBS, CSE or 3-MST have a profoundly reduced resistance to ischemia/reperfusion injury in several organs [[13], [14], [15]]. Hence, tight regulation of endogenous H_2_S seems to be critical for maintenance of renal homeostasis, through different mechanisms that affect both filtration, endocrine and metabolic functions of the kidney. This specific spatiotemporal regulation of renal H_2_S signaling is achieved by regulation of substrate and cofactor availability as well as modulation of the levels, enzymatic activity and localization of the H_2_S-producing enzymes. In the kidney, H_2_S is primarily synthesized by CBS and CSE. While both CBS and CSE are predominantly expressed in proximal tubules [16,17], CSE is also expressed in the glomerulus [18,19]. As compared to CBS and CSE, levels of 3-MST and DAO in the kidney are much lower and their precise role in renal physiology remains unclear [20,21].

In this review, we describe the (sub)cellular and temporal regulation of H_2_S in the kidney and how H_2_S exerts its effects within different organelles such as the nucleus, ER and mitochondria. Moreover, we review how subcellular H_2_S anabolism and catabolism has affects renal (patho)physiology. Next to this, post-translational modification of protein cysteine residues via persulfidation also hold great promise to explain the beneficial properties of H_2_S with potential relevance for the treatment of renal-related diseases, such as hypertension, ischemia/reperfusion and acute kidney injury. Therefore, modulation of the levels, enzymatic activity or localization of H_2_S-producing enzymes could be pharmacologically exploitable targets to modulate endogenous levels of H_2_S.

## 2 Regulation of H_2_S production

### 2.1 Subcellular enzyme localization affects spatial specificity of H_2_S levels

One mode of regulation of H_2_S signaling specificity consists of the multiple H_2_S-producing enzymes that have both unique as well as redundant functions. Regulation of H_2_S production on the organelle level is achieved by (trans)localization of the H_2_S-producing enzymes in the cell. Natively, CBS and CSE reside mostly in the cytosol, while 3-MST mostly resides in the mitochondrion. Translocation of these enzymes into different organelles allows for subcellular control of H_2_S levels, as each enzyme has specific cellular localization signals and cues. CBS has a C-terminal mitochondrial targeting sequence which is recognized by Hsp70 under hypoxic conditions, while CSE requires the mitochondrial outer membrane transporter protein Tom20 to translocate to the mitochondrial lumen upon treatment with the ionophore calcimycin [22,23]. Specificity is also achieved by differences in optimal pH of the H_2_S synthesizing enzymes. The optimal pH for H_2_S production by CBS and CSE is pH 8.5–9.0 [24,25], which is closer to the slightly alkaline pH of 8.0 within the lumen of the mitochondria as compared to the pH of 7.0–7.4 of the cytosol [26]. The optimal pH for H_2_S production by 3-MST is 7.4 [27], close to the pH of the cytosol. The optimal pH for H_2_S precursor production by DAO is 8.4 for l-cysteine as a substrate, but 7.4 for d-cysteine as a substrate. CBS and CSE is localized primarily in the cytosol, but also in vesicles, nucleoli [28] and mitochondria. 3-MST is

Localized in mitochondria [29] and in the cytosol [30]. Finally, DAO – contributing to H_2_S production via 3-MST - is localized in mitochondria and peroxisomes [21,31]. Together, not only substrate availability, enzyme production and translocation, but also local pH and substrate-specific pH optima affect enzymatic activity and consequently, H_2_S levels.

### 2.2 H_2_S production is regulated through substrate and cofactor availability

**Fig. 1:**
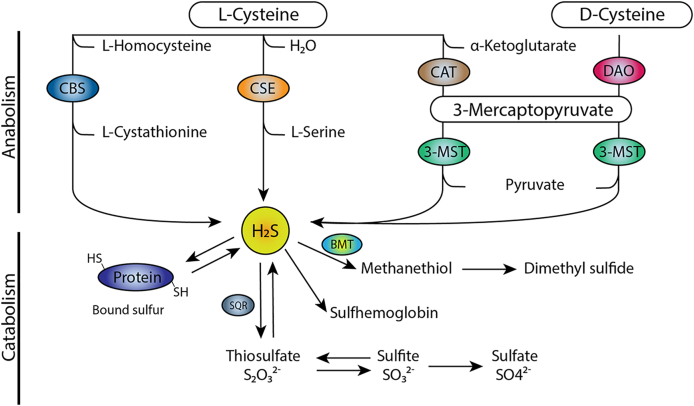
**Metabolism of H**_**2**_**S.** In the canonical pathway of H_2_S production, l-homocysteine is converted to cystathionine by CBS, which is then converted to l-cysteine by CSE. H_2_S is produced from l-cysteine by both CBS and CSE. A second pathway is the production of H_2_S by conversion of d-cysteine or α-ketoglutarate to 3-mercaptopyruvate by DAO and CAT respectively, which is subsequently converted to H_2_S by 3-MST. H_2_S catabolism occurs through persulfidation of proteins, via the formation of sulfane sulfur species, such as thiosulfate via SQR. Catabolism also occurs through the production of methyl mercaptan, also known as methanethiol, to form dimethyl sulfide. A non-enzymatic pathway allows H_2_S to bind to methemoglobin to form sulfhemoglobin.

Spatiotemporal regulation of H_2_S is also achieved by modulating substrate availability for H_2_S production, of which the most important are l-cysteine, l-homocysteine and 3-mercaptopyruvate, and to a lesser extent l-cystine and d-cysteine [32]. The canonical pathways of H_2_S synthesis are depicted in Fig. 1, with l-cysteine and l-homocysteine as major substrates for CBS and CSE. The intracellular concentration of l-cysteine is controlled by a number of independent processes: uptake from plasma in endothelial cells, proteolysis (increasing l-cysteine availability), the transsulfuration pathway (l-cysteine as a substrate for H_2_S synthesis) and the rate of incorporation into glutathione (decreasing or suspending l-cysteine availability) [33]. l-cystine is taken up via the glutamate/cystine antiporter which is under positive control of H_2_S, which forms a positive feedback loop for H_2_S production [34]. Dihydrolipoic acid (DHLA) is a potent anti-oxidant derived from dietary α-lipoic acid (LA), which - by reducing l-cystine - was demonstrated to release free l-cysteine [35] to be used for H_2_S production. The intracellular concentration of l-homocysteine has not been determined yet, possibly because it is a toxic intermediate which is consumed efficiently by CBS, or because the concentration is below detection limits of current analytical methods. d-cysteine required for DAO comes primarily from metabolism of nutrients in the gastrointestinal tract [21]. CBS and CSE require the cofactor pyridoxal-5-phosphate (PLP), also known as vitamin B_6_, to produce H_2_S. H_2_S production by 3-MST depends on thioredoxin (Trx) and dihydrolipoic acid: two important redox balance-maintaining molecules[36]. In turn, the expression of Trx is regulated by transcription factors involved in the antioxidant response, such as Nrf2 [37]. The systems that regulate substrate and cofactor availability are complex, with positive and negative feedback loops, and are coupled to maintenance of the redox balance. Thus, substrate, cofactor availability and H_2_S-producing enzyme abundance control H_2_S anabolism.

### 2.3 H_2_S clearance is achieved both enzymatically and non-enzymatically

While substrate availability, enzyme levels and subcellular localization contribute to spatiotemporal regulation of local H_2_S levels through affecting its synthesis, catabolism, exhalation and excretion regulate lowering of H_2_S levels. Exhalation accounts for <1% of H_2_S elimination in the body, as excretion is the major route responsible for clearance of H_2_S [38]. Before H_2_S can be excreted, it first needs to be converted catabolically. Catabolism of H_2_S occurs both enzymatically and non-enzymatically, of which enzymatic catabolism represents the most important catabolic pathway [3]. In mitochondria, H_2_S is catabolized by the sulfide quinone oxidoreductase system (SQR) to form SQR-bound cysteine persulfides [39,40]. SQR-bound cysteine persulfide is then catabolized into thiosulfate, which is reversible and offers an endogenous donor pool capable of ameliorating hypertensive renal disease and diabetes [41,42]. Furthermore, SQR-bound cysteine persulfide is catabolized into sulfite and sulfate that are excreted via urine. H_2_S is catabolized to sulfite through ethylmalonic encephalopathy 1 protein (ETHE1), which resides in the mitochondrial matrix [43]. Successive methylation of H_2_S by thiol S-methyltransferase (TMT) and thioether S-methyltransferase (TEMT) forms the trimethylsulfonium ion, which is also excreted via urine [44]. Lastly, H_2_S binds to methemoglobin to form sulfhemoglobin in erythrocytes, which act as a sink of H_2_S upon erythrocyte sequestration to the spleen[45].

### 2.5 Intermediates of H_2_S metabolism allow for delayed H_2_S signaling by storage-and-release

H_2_S metabolism is not always a one-way street, as certain catabolic intermediates allow for suspended release of H_2_S. Three different pools of H_2_S have been proposed so far: sulfane sulfur species, acid labile H_2_S and polysulfides. First, H_2_S can be stored in the form of sulfane sulfur species: compounds that contain a sulfur atom with six valence electrons that is bound to another two or more sulfur atoms (RS-Sn-SR) such are: persulfides (RSSH), polythionates (^−^SO_3_-S_n_-SO_3_^-^), organic polysulfanes (HSS_n_SH, RSSS_n_R, RSS_n_SH) and thiosulfate (S_2_O_3_^2−^/^−^S-SO_3_^-^) [5]. H_2_S from sulfane sulfur compounds can be released in reducing conditions or by the activity of thioredoxin/thioredoxin reductase which is a key player in catabolism or sulfane sulfur, mostly incorporated in protein persulfides [46,47]. The only enzyme reported to produce sulfane sulfur compounds from H_2_S is 3-MST, but the mechanism remains unknown [48,49]. Like H_2_S, sulfane sulfur species also possess strong antioxidant capacities [50], which contributes to the prolonged cytoprotective effect of H_2_S and sulfane sulfur production by 3-MST. Second, H_2_S can be stored in the form of acid-labile sulfur that can be released under acidic conditions (pH < 5.4), usually in the iron-sulfur center of mitochondrial enzymes [51,52]. Third, H_2_S can also be stored in the form of polysulfides, facilitated by the enzymes 3-MST and cysteine aminotransferase (CAT) [53]. Delayed H_2_S signaling is achieved through release from H_2_S pools under specific redox conditions. Together, H_2_S anabolism is tightly controlled by enzyme localization, substrate specificity and optimal pH levels, while enzymatic and non-enzymatic metabolism of H_2_S lead to functional intermediates and ultimately, excretion of H_2_S derivatives.

### 2.6 H_2_S signaling in renal physiology

#### 2.6.1 Vasoactive effects of H_2_S affect systemic blood pressure and renal blood flow

Production of H_2_S in endothelial cells governs hormetic (i.e. biphasic) dose-dependent vasoactive effects by influencing endothelial and vasomotor function. Endogenous H_2_S in endothelial cells is produced by CSE[54], 3-MST[55] and CBS [56] and leads to endogenous concentrations of H_2_S in arterial blood in the range of 0.1–1.0 μM [57,58]. In short-term experiments, exogenous NaHS administration induces vasodilation in isolated human mesenteric arteries and rat thoracic aorta [59,60]. In rats, a bolus injection of H_2_S transiently decreased blood pressure, indicating vasodilation. In CSE knockout mice, an increased blood pressure as compared to wild-type mice was observed, indicating a lack of H_2_S-mediated vasodilation [54]. On the contrary, results from a recent study have shown the absence of H_2_S-mediated hypertension in CSE^−/-^ mice as well as the increased level of endogenous NO compared to the CSE wildtype animals. This observation emphasizes the direct chemical reaction between H_2_S and NO where NO reacts with H_2_S as well as their mutual contribution in regulation of vascular tone [61]. Administration of a NaHS, a H_2_S donor in vivo resulted in an increased blood pressure at 10 μmol kg^−1^ min^−1^, while 25 μmol kg^−1^ min^−1^ NaHS led to a decrease in blood pressure [62]. This demonstrates the hormetic effects of H_2_S, conceivably through inhibition of endothelial NO synthase [62].

Tubular function is regulated by H_2_S through modulation of the renal blood flow (RBF) and consequently, glomerular filtration rate (GFR). An increase in tubular H_2_S levels stimulates diuresis, natriuresis and kaliuresis by inhibiting the Na^+^/K^+^/2Cl^-^ cotransporter (NKCC) in chronically salt-loaded rats [63,64]. Both the H_2_S donor NaHS and the H_2_S precursor l-cysteine increase GFR in a dose-dependent manner in rat, which was abolished by concomitant inhibition of CBS and CSE. Inhibition both CBS and CSE alone (by AOAA and PPG, respectively) results in decreased H_2_S levels and a decrease in tubular function [63]. Interestingly, in mice with acute bilateral renal ischemia, addition of NaHS accelerated regeneration of damaged tubular cells, while administrating PPG slowed their regeneration [65]. Hence, generation of endogenous H_2_S in renal endothelial cells leads to a reduced blood pressure, while increasing glomerular filtration rate and tubular function.

#### 2.6.2 Modulation of gene expression by H_2_S is relevant for blood pressure regulation

The spontaneously hypertensive rat (SHR) model was developed by selectively breeding naturally hypertensive rats [66]. The onset of hypertension is associated with a decreased CSE activity in arteries, as demonstrated in thoracic aorta [67]. Further, plasma levels of H_2_S are reduced prior to onset of hypertension, while administration of NaHS (partially) precludes the onset of hypertension [68]. As low levels of endogenous H_2_S induce vasodilation and the lack of CSE is associated with hypertension in mice, it is likely that the reduced levels of H_2_S play a role in the pathophysiology of hypertension in the SHR model. In addition, administration of NaHS in the SHR model downregulates the expression of important components of the RAS system, including renin (Ren), angiotensinogen (Agt), angiotensin-converting enzyme (Ace) and angiotensin II receptor, type 1a (Agtr1a) to levels below those observed in normotensive control rats [69]. Similarly, administration of NaHS downregulates Ren expression and reduces plasma renin levels in the two-kidneys-one-clip (2K1C) rat model for hypertension [70]. In the 2K1C rat model hypertension is induced by temporarily restricting blood flow to one kidney, which activates the renin-angiotensin axis and induces hypertension [71]. In a mouse model of hypertension induced by treatment with angiotensin II, reduced levels of miR-129 (an epigenetic regulator) is associated with an inflammatory response [72]. Treatment with GYY4137 (an H_2_S releasing molecule) restored miR-129 expression to normal, thereby mitigating renal inflammation. Potentially, gene expression changes are governed by epigenetic regulation by H_2_S. Maternal hypertensive rats treated with NaHS produce offspring with an increased methylation of the Agtr1b (angiotensin II receptor, type 1b) and decreased levels of the angiotensin II receptor AT_1_R [73]. Thus, H_2_S affects the expression of genes (for example Ren, Agt and Agtr1a) that play essential roles in blood pressure regulation, which is potentially mediated through epigenetic regulation, as well as miRNA expression.

#### 2.6.3 H_2_S protects against kidney injury via protein persulfidation

**Fig. 2:**
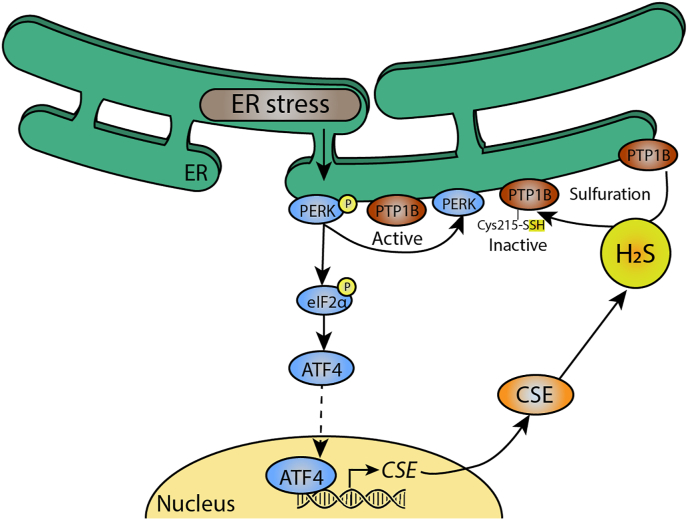
**PTP1B persulfidation attenuates ER stress.** In endoplasmic reticulum stress, a condition caused by accumulation of mis- or unfolded proteins, protein kinase R-like endoplasmic reticulum kinase (PERK) is phosphorylated. This renders protein tyrosine phosphatase 1B (PTP1B) active, which contributes to ER stress. PERK phosphorylation also leads to translocation of the transcription factor Activating transcription factor 4 (ATF4), which enhances expression of CSE. CSE then produces H_2_S, which persulfidate active PTP1B at Cys215, rendering it inactive and attenuating ER stress.

**Fig. 3:**
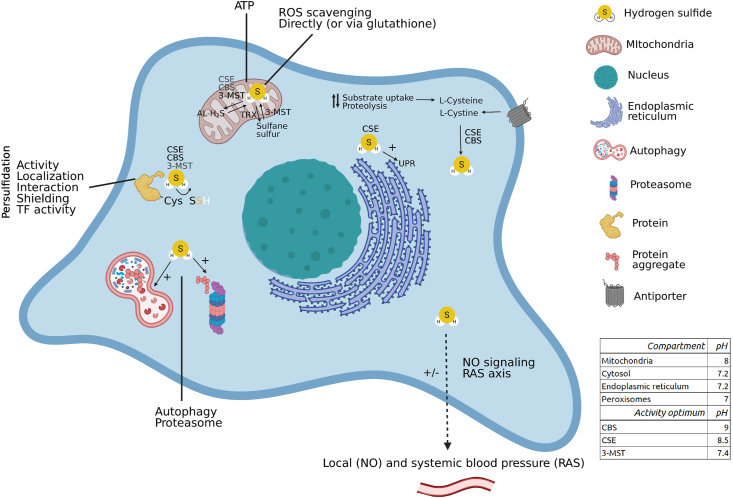
**Renal H**_**2**_**S signaling can be cytoprotective.** Spatiotemporal H_2_S anabolism is regulated by local pH, enzyme optima (table inset), enzyme localization and substrate availability. H_2_S metabolites such as sulfane sulfur or acid-labile pools can reversibly contribute to H_2_S production and signaling. H_2_S signaling regulates renal blood pressure via interaction with NO signaling, and systemic blood pressure by regulating gene expression of components of the renin-angiotensin system. ROS species are directly and indirectly (via glutathione and others) scavenged by H_2_S. At low concentrations H_2_S is an alternative electron donor for oxidative phosphorylation, maintaining ATP production in hypoxic conditions. Further, cysteine persulfidation by H_2_S modulates protein activity, localization, protein-protein interactions, transcription factor activity and protects cysteine moieties from detrimental post-translational modifications. During proteotoxic stress, H_2_S activates the UPR, modulates autophagic flux and proteasome activity. Thereby renal H_2_S signaling is cytoprotective, and contributes to renal functioning. AL-H_2_S: acid-labile H_2_S, OxPhos: oxidative phosphorylation, RAS axis: renin-angiotensin system axis, TF activity: transcription factor activity, TRX: thioredoxin, UPR: unfolded protein response. Synthesizing enzymes are shown if known, bolder characters indicate more experimental evidence. When no enzyme is mentioned, studies were performed with H_2_S donors or the producing enzyme is simply not known. Figure created with BioRender.com.

Persulfidation, also known as “S-sulfhydration”, represents the oxidative modification of a cysteine sulfhydryl group where another thiol moiety (originating from H_2_S, H_2_S donors or another persulfide) is covalently attached to the corresponding cysteine sulfhydryl group ultimately forming the persulfide [5,74] (Fig. 2). Persulfidation modifies protein function and alter protein-protein interactions. First, persulfidation can either increase or decrease function and activity of target proteins. Persulfidation of cysteine residue C150 of mitochondrial glyceraldehyde 3-phosphate dehydrogenase (GAPDH), increases its enzymatic activity [74], while persulfidation on C156 or C152 leads to a profound decreased activity [75]. Second, persulfidation can also alter protein-protein binding kinetics and thereby protein localization, as is the case in the transcription factor Nrf2. Persulfidation of C150 of cytosolic Keap1 initiates dissociation of the bound transcription factor Nrf2 and allows translocation of Nrf2 to the nucleus and enhancement of expression of genes coding for proteins of the antioxidant stress response [76]. Nrf2 has been implicated to protect kidney injury after experimental IR in several studies [77,78]. Finally, persulfidation also protects proteins against detrimental post-translational modifications, such as S-nitrosylation or oxidation during nitrosative and/or oxidative stress conditions [79]. Persulfidation mostly occurs on protein cysteine residues, protecting this moiety from being oxidized by ROS, thus protecting protein function. S-nitrosylation of GAPDH at the aforementioned C150 nullifies enzymatic activity, leading to a marked decrease in ATP production [80]. By persulfidation of GAPDH, but also ATP5a, H_2_S can rescue ATP production. It is known that in AKI, maintaining ATP production is crucial for proper recovery of renal function [81]. Together, H_2_S safeguards renal function after injury through protein persulfidation (see Fig. 3).

#### 2.6.4 H_2_S modulates redox homeostasis, sodium excretion and blood pressure through protein persulfidation

In the kidney, persulfidation of proteins regulate blood pressure and renal sodium handling. Persulfidation of the endothelial growth factor receptor (EGFR) cysteine residues C797/C798 induces endocytosis of the Na^+^/K^+^-ATPase, resulting in loss of function of the Na^+^/K^+^-ATPase in renal tubular epithelial cells [64]. Through these mechanisms, H_2_S induces water and sodium excretion in rats, decreasing blood pressure [64]. Further, persulfidation of the angiotensin II receptor, AT_1_R, by NaHS or l-cysteine attenuates hypertension in angiotensin II-induced hypertensive mice [82]. Thus, protein persulfidation induced by H_2_S influences protein activity, but can also modulate gene expression by affecting protein-protein interactions and expression of miRNAs. The net effect of these layers of signaling are unclear, but are imperative in maintaining redox homeostasis as well as regulation of renal sodium handling and blood pressure.

#### 2.6.5 H_2_S maintain scellular homeostasis upon proteotoxic stress by modulating autophagy

Misfolded proteins are potentially toxic through ER stress and excessive cell death, as is the case in acute kidney injury [83]. Damaged organelles and misfolded proteins are cleared by (macro)autophagy, which is modulated by H_2_S. Exogenous H_2_S (NaHS) inhibits autophagy in neonatal rat cardiomyocytes in an in vitro hypoxia-reoxygenation (HR) model, via PI3K/GSK3β signaling [84] and also in hepatocellular carcinoma (HCC) cells, as illustrated by downregulation of genes in the PI3K/AKT/mTOR pathway[85]. In contrast, in a rat model of ischemia-reperfusion (IR), NaHS addition upregulates genes in the AMPK/mTOR pathway, thereby promoting autophagy and protecting against IR injury [85,86]. It is conceivable that under normal circumstances, H_2_S signaling keeps the rate of autophagy within physiological bounds, while under the circumstances of severe proteotoxic stress, H_2_S can highly increase autophagic flux. By protecting cells from IR injury via modulating autophagy, H_2_S signaling attenuates kidney injury after IR.

#### 2.6.6 H_2_S attenuates proteotoxic stress through protein persulfidation

The endoplasmic reticulum (ER) is indispensable for synthesis, folding, post-translational modification and transport of proteins, and is the first line of defense in protein folding defects [87]. In addition to regulating autophagy, H_2_S can both induce and inhibit proteasomal degradation of proteins to attenuateproteotoxic stress in response to a sustained unfolded protein response (UPR). The proteasome selectively degrades misfolded, ubiquitin-tagged proteins, with Nrf2 as a regulator of the UPR [88,89]. Treatment with Na_2_S partly rescues ischemia-induced heart failure in wild type mice, but not in mice lacking Nrf2. Presumably, effects of Na_2_S are mediated by inducing translocation of Nrf2 after persulfidation of Keap1, as described above [76]. Thus, H_2_S enhances cardiac proteasome activity and thereby attenuates ER stress with cytoprotective effects [90]. On the other hand, in human umbilical vein endothelial cells (HUVECs), H_2_S prevents the proteasomal degradation of eNOS via persulfidation of eNOS [91]. Notably, proteotoxic stress upregulates CSE at low concentrations of doxorubicin and H_2_O_2_ in H9c2 myoblasts, while the expression of CSE decreases upon exposure to higher concentrations of doxorubicin and H_2_O_2_. Addition of NaHS, but also N-acetylcysteine (NAC), rescues cell viability and diminishes ROS accumulation through rescuing CSE expression and H_2_S production [92]. In line with these results, addition of NaHS reduced proteotoxic stress induced by formaldehyde in PC12 cells, but also in nucleus pulposus (NP) cells challenged with IL-1β [93]. Thus, H_2_S is both capable of enhancing the entire proteasomal degradation system, but at the same time prevents specific proteins from being degraded by the very same proteasomal degradation system. Next to modulation autophagy and proteasome activity, H_2_S reduces proteotoxic stress by upregulation protective signaling routes by persulfidation of specific transcription factors.

#### 2.6.7 H_2_S attenuates proteotoxic stress by persulfidation of epigenetic modifiers and transcription factors

Addition of NaHS reduces proteotoxic stress and rescues cell viability presumably through upregulation of silent mating type information regulator 2 homolog 1 (SIRT-1) [94]. Increased activity of SIRT-1 can occur through persulfidation [95]. An alternative explanation for the increase in SIRT-1 activity is modification of a transcriptional regulator of SIRT-1. As such, persulfidation of NF-κβ, an upstream transcriptional regulator of SIRT-1 [96] leads to increased transcriptional activity of NF-κβ and thereby SIRT-1 [97]. Additionally, H_2_S activates PI3K/Akt, ERK1/2 and ATF4 pathways, for example through persulfidation of proteins in these pathways, which reducesER stress (Fig. 2) [98,99]. Recent studies show that persulfidation of SIRT1 and thereby decreasing its deacetylation activity plays a major role in regulation of its epigenetic function [95]. Thus, H_2_S plays an important role in regulating ER function by modulating proteasome activity and inducing protective pathways upon proteotoxic stress by persulfidation of upstream transcription factors and epigenetic modifiers.

Whether H_2_S alleviates proteotoxic stress by H_2_S within ER or through other mediators is not precisely known yet. However, results obtained in HeLa cells, mouse liver and zebrafish using an ER-targeted H_2_S probe, reveal specific localization of H_2_S to ER [100]. It remains to be studied whether local levels of H_2_S within ER are regulated by specific modulation substrate availability or translocation of H_2_S-producing enzymes into the ER. Interestingly, the different H_2_S-producing enzymes seem to fulfill different roles in alleviating proteotoxic stress. During thapsigargin-induced ER stress in HEK293 cells, the generation of carbon monoxide (CO) inhibits CBS by binding to its heme group, which lowers cystathionine production. As cystathionine is an inhibitor of CSE function, its depletion causes CSE to produce more H_2_S to attenuate proteotoxic stress [101]. Collectively, this evidence suggests that H_2_S is a potent modulator of proteotoxic stress through selective modulation of the proteasome and corresponding protein cysteine residues via persulfidation, leading to upregulation of protective signaling routes.

#### 2.6.8 H_2_S attenuates kidney injury through maintaining mitochondrial function

Maintaining ATP production during IR or in AKI is crucial for renal function and recovery. Most ATP is produced by oxidative phosphorylation in mitochondria, with substrates derived from the citric acid cycle to fuel the electron transport chain. Alternatively, H_2_S can also serve as an electron donor in oxidative phosphorylation. Goubern et al. [102] revealed that H_2_S is a substrate of oxidative phosphorylation at nanomolar concentrations. CBS, CSE and 3-MST either reside in the mitochondrion or can translocate there under specific conditions, to contribute to ATP production directly [103]. As such, hypoxia triggers CBS translocation to mitochondria [22,104]. Mitochondrial CBS levels increase 6-fold within 1 h of hypoxia [22]. Another consequence of hypoxia is suppression of oxygen-dependent mitochondrial catabolism (e.g. CoQ-dependent SQR activity) of H_2_S that leads to accumulation of H_2_S in cells. This observation was recently demonstrated by using mass spectrometry based H_2_S-selective chemical probes in ischemic animal tissue and in the organ preservation model system [105] as well as by using H_2_S-sensitive fluorescent sensor in anoxic cell culture [106].

In hypoxia both CSE and CBS activity are associated with elevated ATP production [104,107]. Blockade of CBS or CSE by AOAA/shRNA or PPG, respectively, abrogates the effects of these enzymes on ATP production [107,108]. Stimulating 3-MST function by adding its substrate 3-mercaptopyruvate, increases intramitochondrial H_2_S levels and stimulates the production of ATP [29]. Apart from electron donation of H_2_S, persulfidation of ATP synthase and GAPDH increases their activity, thereby stimulating ATP production. Mice lacking CSE have profoundly lowered levels of ATP synthase and GADPH persulfides, associated with reduced ATP production [74,103]. Thus, H_2_S can stimulate ATP production by donating electrons and by persulfidation of enzymes involved in mitochondrial ATP production. Furthermore, H_2_S reduces cytochrome *c* very efficiently to stimulate the electron flow through the respiratory chain [106]. The same study shows that both endogenous and exogenously applied H_2_S were able to induce cytochrome *c* dependent protein persulfidation that suppresses the apoptotic response by persulfidation of catalytically active cysteine residue of caspase-9 and inhibiting its pro-apoptotic function. In contrast to low levels of endogenous H_2_S, administration of exogenous H_2_S profoundly reduce mitochondrial function by inhibiting cytochrome *c* oxidase (complex IV) through binding to ferric iron (Fe^3+^), thereby halting aerobic ATP generation at alveolar concentrations of >100 ppm [2]. Presumably through this mechanism, exposure to a concentration of 80 ppm H_2_S can (reversibly) suppress metabolic rate and thereby toxicity in mice[109].

Together, while low levels of (endogenous) H_2_S can stimulate ATP production by donating electrons to the electron transport chain and modulating enzymatic activity, high levels of (exogenous) H_2_S may exert toxic effects by inhibiting cytochrome *c* oxidase. Thus, H_2_S maintains ATP production, crucial for renal function after kidney injury.

#### 2.6.9 H_2_S stimulates antioxidant production, which dampens ROS-induced inflammation

Upon metabolic stress, H_2_S can overcome the deleterious effects of mitochondrial dysfunction by stimulating ATP production and exerting anti-oxidant effects by scavenging free radicals, protecting protein residues from being oxidized through persulfidation, and upregulating anti-oxidant mechanisms. The oxidative stress induced by H_2_O_2_ in *Xenopus laevis* kidney epithelial cells, was abolished by (pre)treatment with NaHS [110]. While H_2_S directly scavenges free radicals - for example peroxynitrite - to form sulfinyl nitrite (HSNO_2_), H_2_S also upregulates important anti-oxidant mechanisms including glutathione, a major antioxidant [111]. H_2_S reduces extracellular cysteine to cystine, followed by cellular uptake by the cystine/glutamate antiporter [34]. Further, H_2_S enhances the activity of γ-glutamyl cysteine synthetase (γ-GCS), one of the two enzymes required to produce glutathione [112,113]. The mechanism by which H_2_S affects the γ-GCS activity is not entirely clear, however, γ-GCS expression and protein levels are not affected by H_2_S and likely, post-translation modification by persulfidation accounts for the higher γ-GCS activity upon H_2_S stimulation. Next to scavenging free radicals and upregulating glutathione levels, H_2_S exerts its protective effects through induction of the antioxidant stress response via Nrf2 [114]. Hence, in addition to stimulating ATP production, H_2_S alleviates the damaging effects of mitochondrial dysfunction by reduction of oxidative stress.

The effects of H_2_S on mitochondrial function may explain its protective effects against renal IR injury, thereby dampening inflammation and reducing structural damage induced by IR [115]. Mice lacking CSE are more prone to acute kidney injury induced by IR, associated with a reduced survival [18]. Expression of inflammatory genes and the release of cytokines are reduced through increased persulfidation of transcription factors such as NF-κβ in renal IR. Therefore, H_2_S acts as an antioxidant through increasing antioxidant glutathione levels and upregulation of the antioxidant stress response. Interestingly, a recent study has demonstrated opposite, pro-inflammatory CSE-dependent effects in a mouse model of acute ischemic kidney injury. Here, decreased cellular damage and reduced levels of pro-inflammatory interleukins and cytokines were observed in CSE^−/-^ mice [116]. The observed difference in experimental results between similar studies may be partly explained by the difference in the genetic background of the animal species and their corresponding phenotypes.

Finally, oxidative stress not only affects cellular homeostasis and cell survival, it also affects renal sodium handling by oxidizing phosphatase and tensin homolog (PTEN), thereby augmenting activity of the epithelial sodium channel (ENaC), which facilitates Na^+^ absorption [117]. The effect of oxidative stress on ENaC is abolished by pretreatment with NaHS [117], potentially by protecting PTEN against oxidation by persulfidating the protein, similar to how H_2_S-mediated persulfidation can prevent S-nitrosylation-induced loss of PTEN enzymatic function [118]. Together, precluding mitochondrial dysfunction (i.e. stimulating ATP generation and lowering oxidative stress) precludes kidney dysfunction and damage induced by oxidative stress.

## 3 Conclusion

The production of H_2_S is tightly controlled, both quantitatively as well as spatially by catabolic and anabolic processes. Factors such as substrate availability, protein abundance, local pH and storage capacity control H_2_S anabolism, while oxygen concentration, enzymatic and non-enzymatic mechanisms control H_2_S catabolism. H_2_S metabolites, such as H_2_S bound as sulfane sulfur or acid-labile pools of H_2_S can still exert signaling functions. H_2_S affects renal blood flow by affecting NO levels and thereby, vascular function, as well as regulation of expression of genes responsible for local and systemic blood pressure and sodium excretion. Regulation of protein production and proteotoxic stress is achieved through modulation of autophagic flux, proteasome activity, clearance of aggregated proteins and the UPR via H_2_S signaling, which is relevant to attenuate AKI. H_2_S is an important antioxidant by directly scavenging free radicals, but moreover, it enhances the cellular antioxidant response by serving as a substrate for glutathione production. By forming protein persulfides, H_2_S modulates enzymatic activity, but also transcription factor activity by affecting protein-protein interactions in the cytosol, which induces translocation of the transcription factor to the nucleus. Under hypoxic conditions, H_2_S maintains ATP synthesis by acting as an alternative electron donor. Given the protective effects of H_2_S upon metabolic stress in the kidney, pharmacological targets of H_2_S may be exploited to treat hypertension, or avert damage during acute kidney injury, occurring during for example ischemia/reperfusion, renal transplantation or sepsis.

## Declaration of competing interest

The authors declare that they have no known competing financial interests or personal relationships that could have appeared to influence the work reported in this paper.
